# Health, Polysubstance Use, and Criminal Justice Involvement Among Adults With Varying Levels of Opioid Use

**DOI:** 10.1001/jamanetworkopen.2018.0558

**Published:** 2018-07-06

**Authors:** Tyler N.A. Winkelman, Virginia W. Chang, Ingrid A. Binswanger

**Affiliations:** 1 Division of General Internal Medicine, Department of Medicine, Hennepin Healthcare, Minneapolis, Minnesota; 2 Center for Patient and Provider Experience, Minneapolis Medical Research Foundation, Minneapolis, Minnesota; 3 Department of Social and Behavioral Sciences, College of Global Public Health, New York University, New York; 4 Department of Population Health, School of Medicine, New York University, New York; 5 Division of General Internal Medicine, Department of Medicine, University of Colorado School of Medicine, Aurora; 6 Institute for Health Research, Kaiser Permanente Colorado, Denver

## Abstract

**Importance:**

Health profiles and patterns of involvement in the criminal justice system among people with various levels of opioid use are poorly defined. Data are needed to inform a public health approach to the opioid epidemic.

**Objective:**

To examine the association between various levels of opioid use in the past year and physical and mental health, co-occurring substance use, and involvement in the criminal justice system.

**Design, Setting, and Participants:**

This retrospective, cross-sectional analysis used the 2015-2016 National Survey on Drug Use and Health to assess the independent association of intensity of opioid use with health, co-occurring substance use, and involvement in the criminal justice system among US adults aged 18 to 64 years using multivariable logistic regression.

**Exposures:**

No opioid use vs prescription opioid use, misuse, or use disorder or heroin use.

**Main Outcomes and Measures:**

Self-reported physical and mental health, disability, co-occurring substance use, and past year and lifetime involvement in the criminal justice system.

**Results:**

The sample consisted of 78 976 respondents (42 495 women and 36 481 men), representative of 196 280 447 US adults. In the weighted sample, 124 026 842 adults reported no opioid use in the past year (63.2%; 95% CI, 62.6%-63.7%), 61 462 897 reported prescription opioid use in the past year (31.3%; 95% CI, 30.8%-31.8%), 8 439 889 reported prescription opioid misuse in the past year (4.3%; 95% CI, 4.1%-4.5%), 1 475 433 reported prescription opioid use disorder in the past year (0.8%; 95% CI, 0.7%-0.8%), and 875 386 reported heroin use in the past year (0.4%; 95% CI, 0.4%-0.5%). Individuals who reported any level of opioid use were significantly more likely than individuals who reported no opioid use to be white, have a low income, and report a chronic condition, disability, severe mental illness, or co-occurring drug use. History of involvement in the criminal justice system increased as intensity of opioid use increased (no use, 15.9% [19 562 158 of 123 319 911]; 95% CI, 15.4%-16.4%; prescription opioid use, 22.4% [13 712 162 of 61 204 541]; 95% CI, 21.7%-23.1%; prescription opioid misuse, 33.2% [2 793 391 of 8 410 638]; 95% CI, 30.9%-35.6%; prescription opioid use disorder, 51.7% [762 189 of 1 473 552]; 95% CI, 45.4%-58.0%; and heroin use, 76.8% [668 453 of 870 250]; 95% CI, 70.6%-82.1%). In adjusted models, any level of opioid use was associated with involvement in the criminal justice system in the past year compared with no opioid use.

**Conclusions and Relevance:**

Individuals who use opioids have complicated health profiles and high levels of involvement in the criminal justice system. Combating the opioid epidemic will require public health interventions that involve criminal justice systems, as well as policies that reduce involvement in the criminal justice system among individuals with substance use disorders.

## Key Points

**Question:**

What is the association between various levels of opioid use and health, co-occurring substance use, and involvement in the criminal justice system?

**Findings:**

In this cross-sectional analysis, individuals who reported any level of opioid use were more likely than individuals who reported no opioid use to have physical and mental health conditions and co-occurring substance use. Involvement in the criminal justice system increased with intensity of opioid use, and any level of opioid use was significantly associated with involvement in the criminal justice system in the past year.

**Meaning:**

Public health interventions that involve criminal justice systems, along with policies that reduce criminal justice involvement among individuals with substance use disorders, are needed.

## Teaser

This study uses data from the US National Survey on Drug Use and Health to examine associations between adult opioid use and self-reported physical and mental health, co-occurring substance use, and involvement in the criminal justice system.

## Introduction

In 2016, one person died from a drug overdose in the United States every 9 minutes on average.^1^ Rising drug deaths are being driven by large increases in opioid-related overdoses. With more than 1 in 3 US adults reporting use of prescription opioids (ie, use of prescription opioids that were obtained legally or illegally), the opioid epidemic is now a national public health emergency,^2,3^ and a public health approach is required to address the crisis.^4,5^

Such an approach requires a comprehensive understanding of the populations affected and the systems in which they are involved. Prior work indicates that individuals who use opioids have complex behavioral health profiles and often pass through the criminal justice system, although much of this work focuses primarily on individuals who use heroin.^6,7,8,9,10,11^ The association between intensity of opioid use (ie, no opioid use vs prescription opioid use, misuse, or use disorder or heroin use) and important characteristics such as physical and mental health, co-occurring substance use, and involvement in the criminal justice system has not been comprehensively defined in a nationally representative sample.

Data stratified by intensity of opioid use are critically important as clinicians, communities, and policy makers grapple with strategies to combat the opioid epidemic. For example, if mental illness and involvement in the criminal justice system increase with intensity of opioid use, then interventions that exclude the justice system are likely to miss the highest-risk individuals. Identifying variation among individuals who use opioids can inform efforts to coordinate prevention and treatment efforts among public health, health care, and criminal justice systems and, ultimately, reduce opioid-related morbidity and mortality.^12^

To clarify the specific needs of individuals who use opioids, we examined the following 3 key domains by increasing intensity of opioid use: health characteristics, co-occurring substance use patterns, and involvement in the criminal justice system. We hypothesized that mental illness, polysubstance use, and involvement in the criminal justice system would increase with increasing intensity of opioid use, independent of sociodemographic differences.

## Methods

### Participants and Data Source

We used data from the 2015-2016 National Survey on Drug Use and Health (NSDUH), a nationally representative, cross-sectional survey of individuals aged 12 years or older living in households or noninstitutional group housing (eg, college dormitories, but not jails or prisons) or with no permanent housing (eg, residence in a shelter). We limited our sample to nonelderly US adults aged 18 to 64 years (herein referred to as *adults*) because more than 97% of individuals involved in the criminal justice system are in this age group.^13^ Our study was exempt from human participants review per the Minneapolis Medical Research Foundation’s policy on publicly available, deidentified data sets. We followed the Strengthening the Reporting of Observational Studies in Epidemiology (STROBE) reporting guidelines for cross-sectional studies (eg, clear variable specification, description of statistical analysis, and reporting 95% CIs).^14^

The NSDUH uses a multistage area probability sample for each US state and the District of Columbia and uses computer-assisted personal interviewing with an interviewer present as well as audio computer-assisted self-interviewing to support confidential and private responses. It is the key source of national and state-level data on prevalence of substance use disorders and mental illness in the United States.^15^ The weighted interview response rate for the 2015 NSDUH was 69.7% and for the 2016 NSDUH was 68.4%. The survey underwent a partial redesign in 2015, which resulted in trend breaks with prior years of data.^15^ Although the NSDUH has collected data on prescription opioid misuse and use disorders for decades, 2015 marked the first year that information regarding prescription opioid use of any kind was collected. This change allowed us to provide the first estimates of health, co-occurring substance use, and involvement in the criminal justice system among US adults with any level of opioid use.

### Opioid Use Variables

We categorized our primary independent variable by increasing intensity of opioid use in the past year. Individuals who reported no prescription opioid or heroin use in the past 12 months were labeled *no opioid use*. Those who reported prescription opioid use, but no misuse, in the past year were labeled *prescription opioid use*. Participants who reported prescription opioid misuse, but not a prescription opioid use disorder, in the past year were labeled as *prescription opioid misuse*. Beginning in 2015, prescription opioid misuse was redefined in the NSDUH as use “in any way a doctor did not direct you to use it, including: using it without a prescription of your own; using it in greater amounts, more often, or longer than you were told to take it; or using it in any other way a doctor did not direct you to use it.”^15^^(p71)^ Individuals who were identified by the NSDUH as having prescription opioid dependence or abuse in the past year were labeled as *prescription opioid use disorder*. The *Diagnostic and Statistical Manual of Mental Disorders*, 4th edition (*DSM-IV*),^16^ was used to determine whether an individual’s response met the criteria for prescription opioid dependence or abuse. Individuals who reported any heroin use in the past year were labeled *heroin use*. Opioid use categories were mutually exclusive and based on an individual’s highest level of opioid use intensity (ie, no use vs prescription opioid use vs prescription opioid misuse vs prescription opioid use disorder vs heroin use). We considered heroin use to be a more intensive level of opioid use than prescription opioid use disorder a priori because current evidence suggests that prescription opioid use is on the pathway to heroin use for most individuals who use heroin.^17,18^

### Sociodemographic Variables

We examined sociodemographic characteristics including age, sex, race/ethnicity, educational attainment, marital status, income as a percentage of the federal poverty level, presence of children in the household, current employment status, and rural or urban residence. Rural or urban status was based on core-based statistical areas, as defined by the US Office of Management and Budget, and Rural/Urban Continuum Codes.^19^

### Health Variables

We estimated a number of health characteristics including self-reported health (excellent, very good, or good vs fair or poor), physical health conditions, disability, mental health, and health insurance status. Physical health conditions were identified if a respondent reported ever having been told he or she had the condition by a health professional. Sexually transmitted infection prevalence was limited to a diagnosis in the past year. Respondents who reported a heart condition, diabetes, chronic obstructive pulmonary disease, hepatitis B or C, kidney disease, current asthma, HIV or AIDS, cancer excluding nonmelanoma skin cancer, or hypertension were labeled as having 1 or more chronic conditions.

Disability was identified using the 6-item set of questions recommended by the US Department of Health and Human Services.^20^ The scale measures disability in terms of vision, hearing, cognition, mobility, activities of daily living (eg, dressing or bathing), and instrumental activities of daily living (eg, visiting a physician’s office or shopping). Respondents who indicated a limitation in any domain were considered to have a disability.

Severity of mental illness (ie, none, mild, moderate, or severe) is based on a score validated by the Substance Abuse and Mental Health Services Administration, and it is determined by weighted logistic regression modeling of items from the K6 Psychological Distress Scale, the World Health Organization Disability Assessment Schedule, suicidal ideation in the past year, major depression in the past year, and age.^15^

### Co-occurring Substance Use Variables

We assessed the use of nonopioid substances in the past 12 months, including nicotine dependence, alcohol dependence or abuse, hallucinogen or inhalant use, methamphetamine use, sedative or tranquilizer use or misuse, stimulant use or misuse, cocaine use, and marijuana use. Nicotine dependence was determined by the Nicotine Dependence Syndrome Scale,^21^ and a diagnosis of alcohol dependence or abuse was based on *DSM-IV* criteria.^16^ Misuse of sedatives, tranquilizers, and stimulants was determined using a definition similar to that for prescription opioid misuse.

### Criminal Justice Variables

Respondents were first asked to report whether they had ever been arrested, not including minor traffic violations. Individuals reporting any lifetime arrest were then asked if they had been arrested in the past 12 months. All respondents were also asked about any history of probation or parole in the past 12 months. Individuals who reported any arrest, parole, or probation in the past 12 months were defined as having recent involvement in the criminal justice system. Individuals who reported an arrest during their lifetime but no recent involvement in the criminal justice system were defined as having distant involvement in the criminal justice system.

Individuals who reported an arrest in the last year were asked about the offense for which they were booked and arrested. We categorized assault, rape, murder, homicide, nonnegligent manslaughter, robbery, and child abuse or abduction as *violent crime*. Our definition was similar to the Federal Bureau of Investigation’s definition of violent crime.^22^

### Statistical Analysis

We compared sociodemographic characteristics across the following 5 different levels of opioid use: no opioid use, prescription opioid use, prescription opioid misuse, prescription opioid use disorder, and heroin use.

The adjusted prevalence of health characteristics and co-occurring substance use (dependent variables) across levels of opioid use (independent variable) was obtained using multivariable logistic regression models and predictive margins, controlling for sociodemographic characteristics. Unadjusted estimates of kidney disease and HIV or AIDS are presented because small sample sizes resulted in imprecise estimates in fully adjusted models. We also did not adjust health insurance status because of its known association with income and employment.

Next, we determined the proportion of individuals in each opioid use category who reported any recent or distant incarceration. We then estimated the association between each level of opioid use and involvement in the criminal justice system using logistic regression, with level of opioid use modeled as a categorical variable. We also assessed whether increasing intensity of opioid use trended toward higher levels of involvement in the criminal justice system using logistic regression models, with level of opioid use modeled as a continuous ordinal variable. We first conducted unadjusted estimates of involvement in the criminal justice system among individuals with prescription opioid use, misuse, or use disorder or heroin use compared with those with no opioid use in the past year. We then sequentially controlled for sociodemographic characteristics, health, and substance use in multivariable models to isolate the independent association between various levels of opioid use and involvement in the criminal justice system. We controlled for co-occurring substance use because use of additional substances is known to contribute to involvement in the criminal justice system.^23^ Finally, among those with a history of arrest, we used fully adjusted logistic regression models (controlling for demographics, health, and co-occurring substance use) to examine the association between various levels of opioid use and violent crimes.

All analyses accounted for the complex survey design of the NSDUH, which allowed for nationally representative inferences. We used Stata MP, version 15.1 (Stata Corp), and considered 2-sided *P* < .05 to be statistically significant. Fewer than 2% of observations were missing data for 1 or more variables. Missing data were handled by using casewise deletion.

## Results

### Sample

Our sample consisted of 78 976 respondents (42 495 women and 36 481 men), representative of 196 280 447 nonelderly US adults in 2015-2016. In our weighted sample, 124 026 842 adults reported no opioid use in the past year (63.2%; 95% CI, 62.6%-63.7%), 61 462 897 reported prescription opioid use in the past year (31.3%; 95% CI, 30.8%-31.8%), 8 439 889 reported prescription opioid misuse in the past year (4.3%; 95% CI, 4.1%-4.5%), 1 475 433 reported prescription opioid use disorder in the past year (0.8%; 95% CI, 0.7%-0.8%), and 875 386 reported heroin use in the past year (0.4%; 95% CI, 0.4%-0.5%).

### Sociodemographic Characteristics

Sociodemographic characteristics of our study population are presented in Table 1. Individuals who reported any level of opioid use were significantly more likely than individuals who reported no opioid use to be white, to be in a lower income bracket, and to have lower educational attainment.

**Table 1.** Sociodemographic Characteristics of Study Population by Level of Opioid Use in the United States, 2015-2016

Characteristic	Weighted % (95% CI)				
	No Opioid Use (n = 50 512)	Prescription Opioid Use (n = 23 452)	Prescription Opioid Misuse (n = 3913)	Prescription Opioid Use Disorder (n = 648)	Heroin Use (n = 451)
Overall	63.2 (62.6-63.7)	31.3 (30.8-31.8)	4.3 (4.1-4.5)	0.8 (0.7-0.8)	0.4 (0.4-0.5)
Male	51.4 (50.8-52.0)	43.1 (42.2-44.1)	54.2 (51.8-56.6)	58.9 (54.5-63.3)	66.9 (60.3-72.9)
Race/ethnicity					
White	58.9 (58.0-59.7)	65.6 (64.4-66.7)	65.8 (63.2-68.4)	72.9 (67.1-78.1)	72.3 (66.1-77.7)
African American	12.1 (11.6-12.6)	13.6 (12.8-14.5)	10.5 (9.2-11.9)	11.7 (8.2-16.4)	11.1 (7.0-17.2)
Hispanic	19.2 (18.6-19.9)	14.2 (13.4-15.0)	17.3 (15.4-19.3)	9.3 (6.0-14.1)	13.2 (9.4-18.2)
Other	9.8 (9.2-10.4)	6.6 (6.1-7.2)	6.4 (5.2-7.9)	6.1 (4.2-8.6)	3.4 (2.0-5.7)
Age, y					
18-25	19.0 (18.5-19.5)	13.8 (13.3-14.3)	26.8 (24.9-28.7)	18.5 (15.6-21.9)	25.0 (20.4-30.2)
26-34	19.8 (19.2-20.3)	18.1 (17.5-18.8)	26.1 (24.3-28.0)	25.1 (20.1-30.8)	38.1 (32.5-44.1)
35-49	30.9 (30.3-31.6)	31.5 (30.5-32.5)	26.1 (24.1-28.1)	33.1 (27.9-38.7)	21.2 (16.2-27.2)
50-64	30.3 (29.5-31.1)	36.6 (35.4-37.8)	21.1 (18.9-23.4)	23.3 (17.8-29.9)	15.7 (10.1-23.7)
Educational level					
<High school	13.0 (12.5-13.4)	11.6 (11.0-12.4)	14.8 (13.0-16.8)	16.9 (13.6-20.8)	21.8 (16.2-28.6)
High school	23.9 (23.4-24.4)	25.8 (24.9-26.8)	25.9 (23.6-28.4)	30.2 (25.4-35.5)	35.0 (28.9-41.6)
Some college	30.1 (29.4-30.7)	35.7 (34.9-36.4)	35.3 (33.1-37.6)	35.7 (30.6-41.2)	35.8 (29.7-42.4)
College graduate	33.1 (32.3-33.9)	26.8 (25.9-27.8)	24.0 (22.0-26.0)	17.2 (12.9-22.4)	7.5 (4.4-12.4)
Married	51.3 (50.6-52.0)	51.4 (50.2-52.5)	36.1 (34.1-38.3)	31.6 (26.0-37.7)	14.3 (10.0-20.0)
FPL, %					
<100	15.7 (15.3-16.2)	17.3 (16.5-18.2)	19.6 (17.8-21.6)	23.5 (18.6-29.2)	33.3 (27.5-39.7)
100-200	18.4 (17.7-19.1)	20.2 (19.5-21.0)	22.3 (20.5-24.3)	21.2 (17.2-25.8)	25.9 (20.8-31.6)
>200	65.9 (65.0-66.7)	62.5 (61.4-63.5)	58.0 (55.5-60.5)	55.3 (48.1-62.3)	40.8 (34.7-47.3)
Children in household	45.0 (44.3-45.6)	45.2 (44.3-46.2)	42.0 (39.9-44.1)	43.3 (37.9-48.9)	29.0 (23.0-36.0)
Employment					
Full-time	60.4 (59.7-61.1)	54.5 (53.5-55.5)	56.0 (53.5-58.4)	47.4 (41.9-53.0)	37.8 (31.9-44.0)
Part-time	14.3 (13.9-14.8)	12.7 (12.2-13.4)	15.7 (14.1-17.4)	14.5 (11.1-18.8)	12.5 (8.6-17.8)
Unemployed	5.4 (5.1-5.6)	5.2 (4.8-5.6)	7.5 (6.5-8.7)	8.9 (6.8-11.6)	22.3 (17.8-27.6)
Other	19.9 (19.4-20.4)	27.6 (26.6-28.6)	20.8 (19.0-22.8)	29.1 (24.1-34.8)	27.4 (22.4-33.2)
Rural residence	13.2 (12.7-13.8)	15.7 (14.9-16.5)	13.3 (11.9-14.9)	16.9 (13.3-21.1)	11.9 (8.4-16.7)

Abbreviation: FPL, federal poverty level.

### Health Characteristics

Although individuals with prescription opioid misuse, prescription opioid use disorder, or heroin use were younger, they had significantly worse self-reported health, more chronic conditions, and higher levels of disability than did individuals who reported no opioid use (Table 2). Prevalence of mild, moderate, and severe mental illness generally increased as intensity of opioid use increased. Most individuals with a prescription opioid use disorder had mental illness. Individuals who reported prescription opioid misuse, prescription opioid use disorder, or heroin use in the past year were most likely to lack insurance.

**Table 2.** Health Characteristics by Level of Opioid Use in the United States, 2015-2016^a^

Characteristic	Weighted % (95% CI)				
	No Opioid Use (n = 50 512)	Prescription Opioid Use (n = 23 452)	Prescription Opioid Misuse (n = 3913)	Prescription Opioid Use Disorder (n = 648)	Heroin Use (n = 451)
Fair or poor health	9.2 (8.8-9.6)	16.4 (15.7-17.0)^b^	15.5 (13.6-17.3)^b^	24.9 (20.1-29.7)^b^	17.6 (12.6-22.6)^b^
STI	1.8 (1.6-2.0)	2.7 (2.4-3.0)^b^	3.9 (3.2-4.6)^b^	7.3 (4.5-10.1)^b^	6.0 (3.5-8.5)^b^
Heart condition	5.0 (4.6-5.5)	8.0 (7.4-8.6)^b^	7.2 (5.8-8.6)^c^	11.1 (6.7-15.4)^c^	7.8 (2.6-13.0)
Diabetes	6.5 (6.1-6.9)	9.9 (9.4-10.5)^b^	7.8 (6.1-9.5)	10.5 (6.2-14.8)^d^	4.4 (1.7-7.0)
COPD	2.0 (1.8-2.2)	4.7 (4.3-5.1)^b^	4.4 (3.5-5.3)^b^	6.7 (4.4-9.0)^b^	7.8 (3.2-12.4)^b^
Hepatitis B or C	1.0 (0.8-1.1)	1.5 (1.3-1.8)^b^	1.9 (1.3-2.5)^c^	4.2 (2.0-6.5)^b^	9.5 (5.7-13.2)^b^
Kidney disease^e^	0.7 (0.6-0.9)	2.3 (2.1-2.6)^b^	1.2 (0.8-1.8)^d^	1.1 (0.4-2.8)	1.1 (0.5-3.5)
Asthma	5.1 (4.9-5.3)	8.3 (7.8-8.8)^b^	7.5 (6.3-8.6)^b^	10.4 (6.6-14.2)^c^	5.3 (2.6-8.0)
HIV or AIDS^e^	0.1 (0.1-0.2)	0.4 (0.3-0.5)^b^	0.3 (0.2-0.6)^c^	0.8 (0.2-2.5)^c^	2.1 (0.7-6.0)^b^
Cancer^f^	2.3 (2.1-2.6)	3.7 (3.3-4.1)^b^	4.0 (2.8-5.3)^c^	2.8 (0.9-4.7)	3.1 (0.3-5.8)
High blood pressure	12.6 (12.1-13.0)	18.3 (17.7-18.9)^b^	17.5 (15.3-19.7)^b^	24.4 (19.2-29.7)^b^	18.5 (11.1-25.9)
Any chronic condition	27.1 (26.4-27.8)	37.7 (36.8-38.5)^b^	35.6 (33.4-37.8)^b^	46.9 (41.6-52.3)^b^	39.6 (33.6-45.6)^b^
Any disability	12.1 (11.6-12.6)	21.4 (20.7-22.1)^b^	22.5 (20.7-24.2)^b^	35.7 (30.0-41.5)^b^	26.0 (22.1-30.0)^b^
Mental illness					
None	84.6 (84.1-85.1)	75.3 (74.5-76.0)^b^	64.4 (62.3-66.6)^b^	42.8 (36.7-48.8)^b^	54.9 (48.5-61.3)^b^
Mild	8.1 (7.7-8.4)	11.7 (11.1-12.3)^b^	14.7 (13.2-16.2)^b^	15.1 (11.0-19.3)^b^	15.2 (10.3-20.0)^b^
Moderate	4.0 (3.8-4.3)	6.7 (6.3-7.1)^b^	9.6 (8.4-10.8)^b^	18.0 (13.4-22.5)^b^	11.8 (8.5-15.0)^b^
Severe	3.2 (3.0-3.5)	6.3 (5.9-6.7)^b^	10.7 (9.2-12.2)^b^	20.6 (16.8-24.5)^b^	14.5 (11.0-17.9)^b^
Uninsured^e^	13.3 (12.9-13.8)	9.4 (8.9-9.9)^b^	17.2 (15.8-18.7)^b^	17.3 (13.4-21.9)^d^	21.2 (16.6-26.6)^b^

Abbreviations: COPD, chronic obstructive pulmonary disease; STI, sexually transmitted infection.

^a^ Estimates are adjusted for race/ethnicity, age, sex, educational level, marital status, income, employment, rural vs urban residence, and presence of children in the household unless otherwise specified.

^b^ *P* < .001 compared with no opioid use.

^c^ *P* < .01 compared with no opioid use.

^d^ *P* < .05 compared with no opioid use.

^e^ Estimates are unadjusted.

^f^ Excluding nonmelanoma skin cancer.

### Co-occurring Substance Use

Co-occurring substance use among adults with varying levels of opioid use is described in Table 3. In general, as intensity of opioid use increased, the likelihood of using a given substance increased. Most individuals with prescription opioid misuse, prescription opioid use disorder, or heroin use in the past year reported use of 1 or more additional substances. More than 50% of individuals with a prescription opioid use disorder or heroin use also reported using or misusing sedatives or tranquilizers. Among those with heroin use in the past year, 775 025 of 875 386 (88.5%; 95% CI, 83.3%-92.3%) reported prescription opioid use in the past year.

**Table 3.** Co-occurring Substance Use Patterns by Level of Opioid Use in the United States, 2015-2016^a^

Co-occurring Substance Use	Weighted % (95% CI)				
	No Opioid Use (n = 50 512)	Prescription Opioid Use (n = 23 452)	Prescription Opioid Misuse (n = 3913)	Prescription Opioid Use Disorder (n = 648)	Heroin Use (n = 451)
Nicotine dependence	7.0 (6.7-7.3)	10.3 (9.7-10.8)^b^	15.8 (14.2-17.5)^b^	31.8 (26.7-37.0)^b^	38.3 (30.9-45.7)^b^
Alcohol dependence or abuse	5.9 (5.6-6.1)	7.7 (7.2-8.2)^b^	19.2 (17.7-20.7)^b^	25.6 (21.1-30.2)^b^	16.2 (11.9-20.4)^b^
Other drugs used, No.^c^					
≥1	24.2 (23.6-24.9)	45.5 (44.6-46.4)^b^	68.0 (65.9-70.2)^b^	82.7 (78.5-86.9)^b^	92.9 (89.4-96.4)^b^
1	18.4 (17.9-18.9)	32.6 (31.7-33.5)^b^	31.4 (28.7-34.1)^b^	31.0 (25.4-36.6)^b^	19.6 (14.0-25.2)^b^
2	4.2 (3.9-4.4)	10.0 (9.4-10.6)^b^	20.5 (18.5-22.5)^b^	28.7 (23.2-34.2)^b^	30.0 (23.7-36.4)^b^
≥3	1.7 (1.5-1.8)	2.9 (2.7-3.2)^b^	14.8 (13.4-16.3)^b^	22.0 (18.6-25.4)^b^	40.9 (35.5-46.2)^b^
Sedatives or tranquilizers					
Use	8.9 (8.5-9.3)	26.3 (25.6-27.1)^b^	22.9 (20.9-24.9)^b^	28.6 (23.1-34.1)^b^	28.9 (23.4-34.5)^b^
Misuse	1.4 (1.3-1.5)	2.5 (2.3-2.7)^b^	20.5 (19.0-22.1)^b^	36.1 (30.7-41.4)^b^	30.3 (24.8-35.8)^b^
Stimulants					
Use	3.4 (3.2-3.6)	7.6 (7.1-8.0)^b^	9.1 (7.9-10.3)^b^	13.1 (9.4-16.9)^b^	10.0 (5.8-14.2)^b^
Misuse	1.7 (1.5-1.8)	2.1 (1.9-2.3)^d^	10.2 (9.2-11.3)^b^	15.8 (13.0-18.6)^b^	14.6 (11.1-18.2)^b^
Hallucinogen or inhalant use	1.9 (1.7-2.0)	2.2 (2.0-2.5)^d^	9.1 (8.1-10.0)^b^	9.2 (6.9-11.6)^b^	13.8 (10.4-17.3)^b^
Methamphetamine use	0.4 (0.3-0.4)	0.6 (0.4-0.7)^d^	3.0 (2.4-3.6)^b^	5.1 (3.5-6.7)^b^	14.0 (10.0-18.0)^b^
Cocaine use	1.5 (1.3-1.6)	2.1 (1.9-2.4)^b^	9.1 (7.9-10.4)^b^	12.9 (10.1-15.7)^b^	37.4 (32.1-42.6)^b^
Marijuana use	13.2 (12.7-13.7)	18.3 (17.7-19.0)^b^	40.1 (38.1-42.2)^b^	39.9 (35.4-44.4)^b^	58.0 (50.9-65.0)^b^

^a^ All estimates are adjusted for race/ethnicity, age, sex, educational level, marital status, income, employment, rural vs urban residence, and presence of children in the household.

^b^ *P* < .001 compared with no opioid use.

^c^ Use of sedatives or tranquilizers, stimulants, hallucinogens or inhalants, methamphetamine, cocaine, or marijuana.

^d^ *P* ≤ .01 compared with no opioid use.

### Involvement in the Criminal Justice System

Recent and distant involvement in the criminal justice system among individuals with varying intensities of opioid use are depicted in the Figure. Among individuals with no opioid use in the past year, 19 562 158 of 123 319 911 (15.9%; 95% CI, 15.4%-16.4%) reported any history of involvement in the criminal justice system vs 13 712 162 of 61 204 541 (22.4%; 95% CI, 21.7%-23.1%) of those with prescription opioid use in the past year, 2 793 391 of 8 410 638 (33.2%; 95% CI, 30.9%-35.6%) of those with prescription opioid misuse in the past year, 762 189 of 1 473 552 (51.7%; 95% CI, 45.4%-58.0%) of those with prescription opioid use disorder in the past year, and 668 453 of 870 250 (76.8%; 95% CI, 70.6%-82.1%) of those with heroin use in the past year. Recent involvement in the criminal justice system was reported by 3 601 648 of 123 319 911 (2.9%; 95% CI, 2.7%-3.1%) of individuals with no opioid use in the past year compared with 2 664 068 of 61 204 541 (4.4%; 95% CI, 4.1%-4.7%) of those with prescription opioid use in the past year, 864 626 of 8 410 638 (10.3%; 95% CI, 9.1%-11.5%) of those with prescription opioid misuse in the past year, 286 786 of 1 473 552 (19.5%; 95% CI, 15.5%-24.1%) of those with prescription opioid use disorder in the past year, and 369 519 of 870 250 (42.5%; 95% CI, 36.2%-49.0%) of those with heroin use in the past year.

**Figure.**
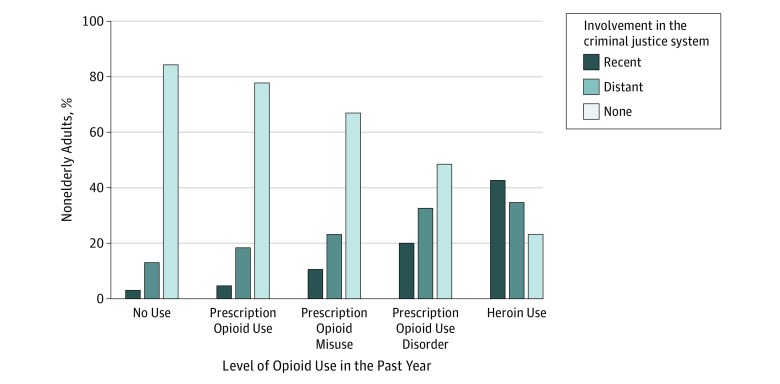
Criminal Justice Involvement by Level of Opioid Use in the United States, 2015-2016 *Recent involvement in the criminal justice system* refers to involvement within the past 12 months. *Distant involvement in the criminal justice system* refers to involvement within an individual’s lifetime but not within the past 12 months.

In unadjusted logistic regression models (model 1), we found that any intensity of opioid use was significantly associated with involvement in the criminal justice system in the past year compared with no opioid use (Table 4). Odds ratios (ORs) increased monotonically and were significantly different in pairwise comparisons. In our trend analysis, involvement in the criminal justice system increased significantly as the level of opioid use increased.

**Table 4.** Involvement in the Criminal Justice System in the Past Year by Level of Opioid Use in the United States, 2015-2016

Characteristic	Odds Ratio (95% CI)^a^			
	Model 1	Model 2	Model 3	Model 4
Past year opioid use				
No opioid use	1 [Reference]	1 [Reference]	1 [Reference]	1 [Reference]
Prescription opioid use	1.5 (1.4-1.7)	1.6 (1.5-1.8)	1.6 (1.4-1.7)	1.4 (1.3-1.6)
Prescription opioid misuse	3.8 (3.3-4.4)	3.0 (2.6-3.5)	2.7 (2.3-3.2)	1.6 (1.3-1.9)
Prescription opioid use disorder	8.1 (6.1-10.7)	6.0 (4.5-8.0)	4.8 (3.6-6.4)	2.2 (1.5-3.2)
Heroin use	24.6 (18.7-32.4)	13.2 (9.7-18.0)	11.3 (8.2-15.5)	4.2 (2.7-6.7)
*P* value for trend	<.001	<.001	<.001	<.001
Controls^b^	None	Demographic factors	Demographic and health factors	Demographic and health factors and substance use

^a^ *P* < .001 for all odds ratios.

^b^ Demographic factors of the 78 976 respondents include race/ethnicity, age, sex, educational level, marital status, income, employment, rural vs urban residence, and presence of children in the household. Health factors include severity of mental illness (none, mild, moderate, or severe), self-reported health, and health insurance status. Substance use includes nicotine dependence, alcohol use disorder, sedative or tranquilizer use and misuse, stimulant use and misuse, cocaine use, marijuana use, hallucinogen or inhalant use, and methamphetamine use.

In model 2, we controlled for sociodemographic differences, and in model 3, we additionally controlled for health factors (severity of mental illness, self-reported health, and health insurance). Similar to model 1, ORs in model 2 and model 3 for prescription opioid use, prescription opioid misuse, prescription opioid use disorder, and heroin use were significantly higher than no opioid use. Again, ORs in model 2 and model 3 increased monotonically and were significantly different in pairwise comparisons. In our final model, model 4, we controlled for co-occurring substance use patterns. Odds ratios for each level of opioid use remained significantly higher than no opioid use in the fully adjusted model. Linear trends remained statistically significant in models 2, 3, and 4, with involvement in the criminal justice system increasing significantly as the level of opioid use increased.

On average, 965 191 of 4 205 904 (23.0%; 95% CI, 21.0%-25.0%) arrests in our cohort were for violent crime. Among those arrested, opioid use was not associated with violent (vs nonviolent) crime relative to no opioid use (prescription opioid use: OR, 1.1; 95% CI, 0.8-1.6; *P* = .59; prescription opioid misuse: OR, 1.4; 95% CI, 0.9-2.3; *P* = .11; prescription opioid use disorder: OR, 0.8; 95% CI, 0.3-2.1; *P* = .69; and heroin use: OR, 1.0; 95% CI, 0.5-2.0; *P* = .93).

## Discussion

Among a national sample of nonelderly adults, physical and mental health conditions, co-occurring substance use, and involvement in the criminal justice system were higher among individuals with any level of opioid use compared with individuals who reported no opioid use. More than half of individuals with a prescription opioid use disorder or heroin use in the past year reported contact with the criminal justice system. We found that, as the level of opioid use increased, involvement in the criminal justice system also increased after accounting for sociodemographic, health, and substance use differences. The large proportion of individuals with opioid use disorders who have physical and mental health conditions and who contact the criminal justice system suggests that public health interventions to combat the opioid epidemic must target populations with involvement in the criminal justice system and should coordinate treatment between the criminal justice and health care systems.

We also provide a detailed characterization of health conditions among individuals with varying levels of opioid use. To our knowledge, prevalence of chronic disease and disability have not been previously reported among individuals with various levels of opioid use. Among individuals with any level of opioid use, the prevalence of chronic disease was higher, partially driven by high rates of chronic obstructive pulmonary disease and hepatitis B or C, and disability was more than twice as common compared with individuals with no opioid use.

Previous research indicates that more than half of prescription opioids are used by individuals with mental health disorders.^9^ We found that at least 1 in 5 individuals with a prescription opioid use disorder or heroin use in the past year had severe mental illness and that the prevalence of severe mental illness generally increased as the level of opioid use increased. We also provide the most recent estimates of co-occurring substance use patterns among individuals who use opioids, which indicate high levels of both nonopioid prescription use and illicit substance use, particularly among those with a prescription opioid use disorder or heroin use.^10,18^ Our findings support prior calls for delivering mental health treatment alongside treatment for opioid use disorders to address co-occurring mental illness and other substance use disorders.^24,25^

To our knowledge, this is the first report to quantify the independent association between recent involvement in the criminal justice system and any level of opioid use. The association between opioid use and involvement in the criminal justice system was partially moderated by sociodemographics, health, and co-occurring substance use but remained significant after accounting for these factors. Co-occurring substance use had a particularly large moderating association among individuals with prescription opioid use disorder or heroin use. The large proportion of individuals with opioid use in the past year who interact with the criminal justice system is problematic because involvement in the criminal justice system interferes with treatment through a variety of mechanisms.^26^ First, most individuals with an opioid use disorder will not receive treatment while they are incarcerated.^27^ Second, individuals who are incarcerated while receiving treatment for an opioid use disorder are often forced to withdraw.^28^ Forced withdrawal reduces the likelihood that an individual will reenter treatment on release.^29^ Finally, only 1 in 20 individuals on community supervision who are referred to treatment ultimately receive first-line therapy.^30^

The overlap we found between involvement in the criminal justice system and opioid use suggests that access to opioid treatment within the criminal justice system is a critical public health issue. Starting or continuing methadone hydrochloride or buprenorphine hydrochloride while incarcerated increases treatment entry and retention on release and reduces postrelease mortality.^12,31,32,33^ Treatment with extended-release naltrexone hydrochloride, a monthly injection that reduces opioid cravings, also reduces relapse among individuals with a history of involvement in the criminal justice system.^34,35^ However, effective implementation of medication-assisted therapy programs within the criminal justice system is challenging owing to stigma, as well as concerns about safety and control.^35,36^ There are also financial barriers to implementation of robust opioid treatment programs.^37^

### Limitations

Our study has several important limitations. First, because of the cross-sectional design of the NSDUH, we are unable to determine when an individual’s opioid use began relative to his or her period of involvement in the criminal justice system; thus, causal mechanisms cannot be elucidated in this cross-sectional study. For example, we cannot determine whether opioid use precedes involvement in the criminal justice system or whether involvement in the criminal justice system leads to opioid use. Second, opioid-related questions were expanded and altered in the 2015 NSDUH. The Substance Abuse and Mental Health Services Administration has indicated that opioid-related prevalence estimates beginning in 2015 are not comparable with prior years, and we are, therefore, unable to examine trends over time.^15^ Third, the NSDUH is the only community-based survey to collect information regarding involvement in the criminal justice system and opioid use, but it does not provide information specifically regarding incarceration or include individuals who are currently incarcerated. Fourth, the NSDUH relies on self-reported symptoms and outcomes, and there may be social desirability to underreport opioid use, which would bias our estimates toward the null.

## Conclusions

In this study, we provide the most comprehensive national estimates to date of health conditions, co-occurring substance use, and involvement in the criminal justice system among individuals with varying levels of opioid use. More than half of individuals with prescription opioid use disorder or heroin use reported a history of involvement in the criminal justice system, and any level of opioid use was independently associated with involvement in the criminal justice system in the past year. Given the complex health and criminal justice profiles of individuals who use opioids, policy makers should carefully consider how changes to public health insurance programs and sentencing guidelines may aid or hinder a public health approach to the opioid epidemic. Future research should examine whether improved linkages between criminal justice, public health, and health care systems can reduce opioid-related morbidity and mortality and further explore the causal pathways involved in the association between opioid use and involvement in the criminal justice system.
